# Distribution of ixodid ticks of cattle and associated risk factors in and around Guder town, west Shewa zone, Oromia, Ethiopia

**DOI:** 10.1016/j.parepi.2025.e00454

**Published:** 2025-08-15

**Authors:** Isayas Asefa Kebede, Kirubel Abreham, Asemamaw Alemayew, Dimamnesh Worku, Tefera Tarekagne, Megersa Akasa Debele, Segni Bedasa Gudina, Abrahim Dawed Ahmed, Haben Fesseha Gebremeskel

**Affiliations:** a School of Veterinary Medicine, Ambo University, P.O. Box 19, Guder, Ethiopia; b Ethiopian Agriculture Authority, Eastern Branch, Dire Dawa, Ethiopia; c School of Veterinary Medicine, Wolaita Sodo University, Wolaita Sodo, Ethiopia

**Keywords:** Cattle, Guder, Identification, Prevalence, Tick

## Abstract

Ticks are the most prominent ectoparasites of cattle, causing significant losses. From March to May 2024, a cross-sectional study was carried out in and around Guder town of the west Shewa zone of the Oromia region, Ethiopia, to identify species of ixodid ticks infesting cattle and estimate their prevalence and associated risk factors. Adult ixodid ticks were collected from 200 cattle using systematic random sampling methods and classified into species based on their morphology using a stereomicroscope. Of the examined cattle, 75.0 % (95 % CI: 68.5–80.6) were infested with one or more tick species. The highest prevalence of tick parasites was recorded in Mamo Mezemir kebele, male, young, extensive management system, and good-body condition of cattle, with prevalences of 94.7 %, 87.5 %, 86.7 %, 77.6 %, and 80.0 %, respectively compared to counterparts. Four genera of ticks were identified viz. *Amblyomma, Rhipicephalus (Boophilus), Hyalomma,* and *Rhipicephalus.* A total of 702 ticks were collected*. Rhipicephalus evertsi evertsi* and *Hyalomma rufipes* were the most (38.7 %) and least (8.1 %) abundant tick species, respectively. The peasant associations and sex were the only risk factors associated with the tick infestations (*p* < 0.05). Cattle in the Mamo Mezemir kebele were 17 (OR = 16.9; 95 %CI: 1.9–149.2) times more likely to be infested by ticks than those in other regions. This study shows that ticks were a most significant concern for cattle in the studied area. Thus, strategic tick control, which includes the use of acaricides, and creating awareness for the livestock owners about the impact of tick infestation is recommended.

## 1 Introduction

Ethiopia has the highest livestock population in Africa, with 65 million cattle, 51 million goats, 49 million chickens, 40 million sheep, and 8 million camels (Central Statistical Agency (CSA), 2020); all of which play an important role in the country's economic development, accounting for 15–17 % of total GDP and 35–49 % of agriculture GDP (CSA, 2019). Livestock is crucial in providing export commodities like meat, live animals, hides, and skins that help the government gain foreign cash. In the highlands of the country, crop-livestock farming is mostly employed for drought power, milk production, and manure (Duguma and Janssens, 2021). However, the contribution of the cattle subsector to the national economy is significantly limited by a variety of factors. The most significant restrictions to cattle productivity are endemic diseases, including parasitic infestation (both external and internal parasites), inadequate veterinary services, and a lack of government attention (Jemal et al., 2021; Duguma and Janssens, 2021).

Ticks are external parasites of livestock categorized (together with mites) in the class Arachnida, order Ixodida (Makwarela et al., 2023). They are classified into three extant families: Ixodidae, Argasidae, and Nuttalliellidae, with a total of 896 species (702 ixodids, 193 argasids, and 1 Nuttalliella) (Guglielmone et al., 2010). Ticks (both ixodids and argasids) develop in four stages: eggs, larvae, nymphs, and adults (Minjauw and McLeod, 2003; Taylor et al., 2015). They cause significant economic losses, both directly through blood-sucking, weight loss, and decreased productivity and indirectly as vectors of many pathogens. The tick saliva can also trigger allergic reactions (Minjauw and McLeod, 2003; Taylor et al., 2015). The pathogens transmitted include protozoa, rickettsia, and viruses of livestock, all of which are economically significant worldwide. Tick-borne protozoan diseases (theileriosis and babesiosis), rickettsial diseases (anaplasmosis and ehrlichiosis), and tick-associated dermatophilosis are major health and management issues for livestock in many developing countries, including Ethiopia (Bekele, 2002; Lefebvre et al., 2010; Tomassone et al., 2012; Kumsa et al., 2015; Hadgu et al., 2018). The most economically important ixodid ticks for livestock in tropical regions belong to the genera *Hyalomma, Rhipicephalus*, and *Amblyomma* (Frans, 2000; Alemu et al., 2014).

Ticks are common in all agro-ecological zones of Ethiopia (Sileshi et al., 2001; Sileshi et al., 2007; Tiki and Addis, 2011; Gedilu et al., 2014*;* Tamerat et al., 2015; Asefa et al., 2017). About 47 tick species have been identified in Ethiopia, mainly *Rhipicephalus evertsi evertsi, Rhipicephalus muhsamae, Rhipicephalus lunulatus, Haemaphisalis leachi leachi, Haemaphisalis parmata, Amblyomma variegatum, Amblyomma lepidum, Amblyomma cohaerens, Amblyomma gemma,* and *Hyalomma marginatum rufipes* (Sileshi et al., 2001; Abera et al., 2010; Jemal et al., 2021). Ticks are primarily controlled using conventional acaricides. However, improper use of these acaricides has adverse effects on host organisms and the environment. Environmental contamination, residues in feed, high costs, residuals in milk and meat, and the development of acaricide resistance in ticks have all prompted research into new safe tick control approaches (Mekonnen, 2001; Johnson, 2023).

Investigating tick species in this study area is crucial for the up-to-date status of ticks and developing more cost-effective tick and tick-borne disease control and eradication programs. As a result, if the common ticks are known to an area and their favored hosts, it is possible to devise an intervention to lower tick load and reduce their impacts, including the disease they are likely to transfer (Tafesse and Amante, 2019). Although several studies have been conducted on ixodid tick infestation in Ethiopia (Tiki and Addis, 2011; Gedilu et al., 2014*;* Tamerat et al., 2015; Asefa et al., 2017*;* Tafesse and Amante, 2019; Wondimu and Bayu, 2021; Lemu et al., 2023), it is still important to generate periodic and up-to-date information about the status of different tick species and associated factors across the country. As a result, diverse research in various sections of the country is currently required to reduce economic losses due to tick infestation. Furthermore, there had been no previous research or published information on tick infestation in cattle in and around Guder town.

Therefore, the objectives of the study were to identify and estimate the prevalence of various tick species, and to assess risk factors that could contribute to tick infestation on cattle in the Toke Kutaye district, west Shewa zone, Ethiopia.

## 2 Materials and methods

### 2.1 Description of study area

**Fig. 1:**
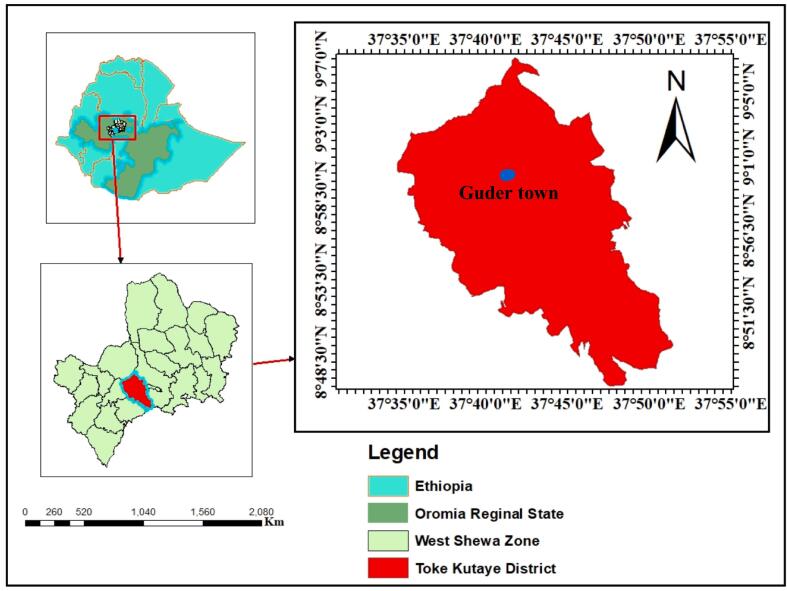
Map of the Guder town in the Toke Kutaye district (**Source:** ArcGIS, 2024).

The study was carried out in Guder town, Toke Kutaye district (Fig. 1). Guder town is located in the west Shewa zone of Oromia regional state, Ethiopia, at 8°49′0“ to 9°5’30” N latitude and 37° 31′30“ to 37°53’15” E longitude. The town lies 126 km west of Addis Ababa. The area's elevation ranges from 1250 to 3200 m above sea level (m.a.s.l). The study area receives an average annual rainfall of 800 to 1100 mm and has an annual mean temperature ranging from 16 °C to 22 °C. Rainfall is bimodal, with a short rainy season from February to May and a long one from June to September. The farming system in the area is a mixed livestock/crop system, with animals playing an important role in the community's livelihood (Abdisa, 2018). Guder town has a total animal population of 145,4384 cattle, 50,413 sheep and goats, 24,772 poultry, and 47,384 horses, mules, and donkeys (Ayana and Fayisa, 2022).

### 2.2 Study animals

The study animals were indigenous and cross-bred cattle of all ages and both sexes, and kept under various management regimes (extensive, intensive, and semi-intensive). The age of the animal was categorized: young ≤3 years and adult >3 years (Lahunta and Habel, 1986), and body condition score: poor, medium, and good (Nicholson and Butterworth, 1986)*.*

### 2.3 Study design

A cross-sectional study was conducted from March to May 2024 to identify species of ixodid ticks infesting cattle and estimate their prevalence as well as associated risk factors in and around Guder town of the Toke Kutaye district, west Shewa zone, Ethiopia.

A multi-stage sampling technique was employed for the selection of the sampling units. From the study district, four peasant associations (PAs): namely Jitu Idris, Mamo Mezemir, Abba Gada, and Gadisa Godfo were selected through purposive sampling methods (based on infrastructures, and livestock population). Then, herds and individual animals in the selected PAs were selected using a systematic random sampling technique. Finally, ticks were collected from half the body of all cattle in selected PAs.

### 2.4 Sample size determination

The sample size was calculated using Thrusfield (2018) formula with 95 % confidence interval and 5 % absolute precision. Using 25.6 % of the prior prevalence reported by Tiki and Addis (2011) of ixodid ticks on cattle in and around Holeta town, the current sample size was determined as follows: *n* = 1.96^2^*Pexp (1-pexp)/d^2^; Where, n = the required sample size, Pexp = expected prevalence*,* and d = desired level of precision (5 %). Consequently, 293 was the minimum sample size. Nevertheless, a total of 200 animals were sampled for the current study due to logistical constraints and study period. In addition, Arsham's (2007) formula was used to determine the minimum number of livestock keepers needed for the survey: *N* = 0.25/SE^2^; Where N = sample size, S = standard error, 5 %. Accordingly, a total of 100 farmers were included in the questionnaire survey.

### 2.5 Data collection

#### 2.5.1 Tick collection and identification

After the selected animals were confined, all visible adult ticks were collected from the half-body regions of cattle. Ticks were carefully removed manually by pulling horizontally on the body surface. The collected ticks were kept in universal bottles containing 70 % ethyl alcohol and labeled with the PAs, age, breed, BCS, husbandry system, and host sex. The specimens were subsequently transported to the Ambo University, Guder Mamo Mezemir Campus parasitology laboratory in screw-cupped bottles. The ticks were then identified and counted to genus and species level with a stereomicroscope using standard identification keys provided by Walker (2003). To calculate whole-body tick burdens, half-body tick counts on cattle were doubled.

#### 2.5.2 Questionnaire survey

Questionnaires were formulated from available literature and by the current researchers, to assess the knowledge and practices of the respondents regarding tick infestation in cattle in the study area. All questionnaires were organized sequentially to facilitate discussion and interpretation. Data was collected through face-to-face interviews using a pre-tested structured questionnaire. The questionnaire was first prepared in English and translated to Afan Oromo and Amharic for appropriateness and easiness in approaching the study participants. For validation of the questionnaire, a sample of 15 randomly selected individuals in the study area who were not included in the main study were interviewed. The questionnaire was assessed for its understandability, clarity, completeness, reliability, and socio-cultural acceptability. Some questions that were unclear to the respondents were revised.

During the study period, 100 farmers from the four purposefully selected *Kebeles* (*Kebele*: The smallest administrative unit) were considered for a questionnaire survey to provide feedback on significant factors that lead to cattle tick infestation problems. Half of the participants were those whose cattle were sampled for ticks and half of them were not. The interviewers were trained before collecting the data (KA, AA, DW, TT). Then, each participant was randomly selected and given 30 min to fill the questions accordingly. The participants were chosen based on their willingness and eligibility based on age (e.g., ≥ 18 years old with adequate communication and understanding skills).

### 2.6 Data management and analysis

The obtained data were exported to a Microsoft Excel spreadsheet (2019) and analyzed using STATA 14^@^ software (StataCorp LP, College Station, TX). The percentage of tick-positive animals was regarded as the prevalence. The relationship between tick infestations (dependent variable) and several independent variables (age, sex, BCS, and PAs) was studied using univariable logistic regression. Multicollinearity was tested for predictors with a liberal *P* value ≤0.25, and covariates with Kruskal gamma values ranging from −0.6 to +0.6 were included in multivariable logistic regression analysis. The final model was developed using log-likelihood and Wald statistics, as well as the stepwise backward exclusion technique. Model fit and validity were verified using Hosmer and Lemeshow statistics as well as the Receiver Operating Curve (ROC) (Dohoo et al., 2003). The significance level was considered when the *p*-value was less than 0.05 for variables with a significant odds ratio (OR) value at 95 % confidence.

### 2.7 Ethical considerations

Ambo University, School of Veterinary Medicine, provided verbal ethical approval for the study, which included the establishment of guidelines. The local community leader (LCL chairman) in each sampled area was informed about the research at the appropriate *kebeles*. At four of the sites, researchers traveled from village to village to collect representative data or samples, and in all cases, owners gave their approval and were present when their cattle were sampled.

## 3 Results

### 3.1 Prevalence of tick infestation in the study area

Of the 200 cattle examined for tick presence, 150 cattle were infested with one or more tick species, with an overall prevalence of 75.0 % (95 % CI: 68.5–80.6). A high prevalence of tick infestation was observed in Mamo Mezemir Kebele at 94.7 % (95 % CI: 69.2–99.3), compared with 67.2 % (95 % CI: 54.1–78.1) for Abba Gada. Likewise, local breeds showed a slightly higher prevalence (75.1 %, 95 % CI: 67.4–81.5) compared to crossbreeds (74.5 %, 95 % CI: 61.2–84.4). The male and young animals had a high prevalence at 87.5 % (95 % CI: 80.5–92.2) and 86.7 % (95 % CI: 75.3–93.2) compared with 52.7 % (95 % CI: 41.2–64.1) and 70.0 % (95 % CI: 61.8–77.0) for females and adult animals, respectively. Also, extensively reared animals showed a higher prevalence (77.6 %, 95 % CI: 69.4–84.1) compared to semi-intensively reared ones (69.6 %, 95 % CI: 56.3–80.3) (Table 1).

**Table 1:** Prevalence of tick infestation on the cattle in the study area.

Variables	Category	NEA	NPA	%	95 %CI
PAs	Abba Gada	58	39	67.2	54.1–78.1
	Jitu Idris	68	55	80.8	69.6–88.6
	Gadisa Godfo	55	38	69.1	55.6*–*79.9
	Mamo Mezemir	19	18	94.7	69.2–99.3
Breed	Local	145	109	75.1	67.4–81.5
	Cross	55	41	74.5	61.2–84.4
Sex	Male	128	112	87.5	80.5–92.2
	Female	72	38	52.7	41.2–64.1
Age	Young	60	52	86.7	75.3–93.2
	Adult	140	98	70.0	61.8–77.0
Management system	Semi-intensive	56	39	69.6	56.3–80.3
	Intensive	19	14	73.6	49.4–88.9
	Extensive	125	97	77.6	69.4–84.1
Body condition score	Poor	143	106	74.1	66.3–80.6
	Medium	37	28	75.7	59.1–87.0
	Good	20	16	80.0	56.4–92.5
Total		200	150	75.0	68.5–80.6

NB: NEA = Number of Examined Animals; NPA = Number of Positive Animals*;* % = Prevalence; CI = Confidence Interval.

### 3.2 Tick species diversity

Three tick genera were identified: *Amblyomma*, *Rhipicephalus (Boophilus)*, *Hyalomma*, and *Rhipicephalus*. A total of 702 ticks were collected, comprising 432 males and 270 females (Table 2).

**Table 2:** Sexwise ratio of identified tick species in the study area.

Tick species	Male	Female	Sex ratio (M: F)
*Amblyomma variegatum*	112	67	1.7:1
*Amblyomma lipidum*	66	22	3:1
*Rhipicephalus (Boophilus) decoloratus*	48	58	0.8:1
*Rhipicephalus evertsi evertsi*	169	103	1.6:1
*Hyalomma rufipes*	37	20	1.9:1

**Fig. 2:**
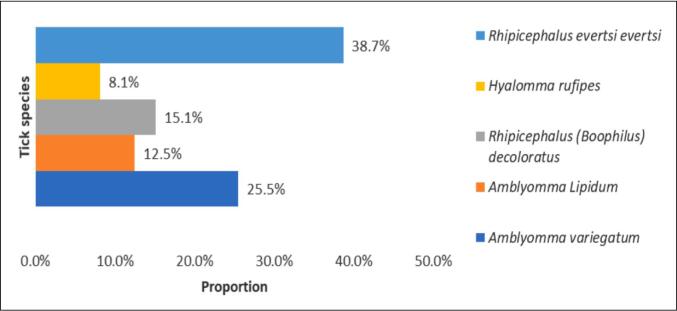
Proportion of tick species identified in the study area.

From the collected tick, the proportion was highest for *R. evertsi evertsi* (38.7 %) and lowest for *H. rufipes* (8.1 %) (Fig. 2).

Ticks were collected from six different body parts: dewlap, under tail, ear, leg, udder, and scrotum. Tick species have various predilection sites, with *A. variegatum* preferring the scrotum and *R. evertsi evertsi* preferring the undertail. Tick species were highest and least in ears (21.8 %) and udder region (1.1 %), respectively (Table 3).

**Table 3:** Distribution of tick species on body regions of cattle in the study area.

Body Region	*Amblyomma variegatum* +ve (counted)	*Amblyomma lipidum* +ve (counted)	*Rhipicephalus (Boophilus) decoloratus* +ve (counted)	*Rhipicephalus evertsi evertsi* +ve (counted)	*Hyalomma rufipes* +ve (counted)	Total	Prevalence
Ears	–	–	–	22 (153)	–	153	21.8
Legs	11 (46)	–	9 (66)	–	–	112	15.9
Scrotum	23 (75)	–	–	–	9 (27)	102	14.5
Udder	14 (58)	–	–	1 (9)	4 (11)	78	1.1
Tail	–	–	–	5 (110)	7 (19)	129	18.4
Dewlap	–	21(88)	3(40)	–	–	128	18.2

NB: +ve = Tick-infested animals.

### 3.3 Risk factors associated with tick infestation

#### 3.3.1 Univariable logistic regression analysis

Univariable logistic regression analyses were performed to assess the strength of the association between predictor factors and tick infestations. There was a significant (p < 0.05) association between tick infestations and predictor factors namely PAs, sex, and age (Table 4).

**Table 4:** Univariable logistic regression analysis of risk factors associated with tick infestations.

Variables	Category	%	OR	95 %CI for OR	p-value
PAs	Abba Gada	67.2	Ref.	**–**	**–**
	Jitu Idris	80.8	2.1	0 *0*.9–4.7	0.082
	Gadisa Godfo	69.1	1.1	0.5–2.4	0.833
	Mamo Mezemir	94.7	8.8	1.1–70.7	0.041
Breed	Cross	74.5	Ref.	–	–
	Local	75.1	1.0	0.5–2.1	0.927
Sex	Female	52.7	Ref.	–	–
	Male	87.5	6.3	3.1–12.6	<0.0001
Age	Adult	70.0	Ref.	–	–
	Young	86.7	2.8	1.2–6.4	0.015
Management system	Extensive	77.6	Ref.	–	–
	Semi-intensive	69.6	0 *0*.8	0 *0*.3–2.6	0.738
	Intensive	73.6	1.2	0.4–3.7	0.706
Body condition score	Poor	74.1	Ref.	–	–
	Medium	75.7	1.1	0 *0*.5–2.5	0.847
	Good	80.0	1.4	0 *0*.4–4.4	0.572

NB:% = Prevalence; OR = Odds Ratio; CI = Confidence Interval; Ref = Reference category; PAs = Peasant association.

#### 3.3.2 Multivariable logistic regression analysis

Potential risk factors (PAs, sex, and age) with *p*-values ≤0.25 were analyzed with multivariable logistic regression utilizing the backward elimination approach, and the final model was developed. Peasant associations and sex were the only risk factors associated with tick infestations, showing statistical significance (p < 0.05). Cattle in the Mamo Mezemir PAs were 17 times (OR = 16.9; 95 % CI: 1.9–149.2) more likely to be infested than that of Abba Gada. Similarly, male cattle were eight times (OR = 8.4; 95 % CI: 3.8–18.6) more likely to be tick-infested than females (Table 3). The Hosmer-Lemeshow goodness-of-fit test indicated that the model suited the data (χ2 = 13.88; *p* = 0.5033) and that multicollinearity did not violate the assumption (AUC = 80.4 %) (Table 5).

**Table 5:** Multivariable logistic regression analysis of risk factors associated with tick infestations.

Variables	Category	%	OR	95 %CI for OR	p-value
PAs	Abba Gada	67.2	Ref.	**–**	**–**
	Jitu Idris	80.8	2.8	1.1–7.5	0.039
	Gadisa Godfo	69.1	1.0	0.4–2.6	0.939
	Mamo Mezemir	94.7	16.9	1.9–149.2	0.011
Sex	Female	52.7	Ref.	–	–
	Male	87.5	8.4	3.8–18.6	<0.0001

NB: % = Prevalence; OR = Odds Ratio; CI = Confidence Interval; Ref = Reference category; PAs = Peasant association.

### 3.4 Knowledge and practices of farmers towards ticks

Seventy-nine percent of respondents identified tick infestation as a problem, and 89 % were aware of tick-borne diseases. Among respondents, 61 % acknowledged the efforts of local veterinary workers to reduce and control tick infestation, whereas 77 % responded that bovine was a highly infested species. Likewise, 58 % of them believed that the dry season favors tick infestation, and 64 % used hot iron to control ticks traditionally (Table 6).

**Table 6:** Knowledge and practices of farmers towards ticks in the study area (*n* = 100).

Variables	Categories	Respondents		%	SE	95 %CI for %
		Male	Female			
Are you familiar with ticks?	Yes	83	17	100	0	–
	No	–	–	–	–	–
Are ticks a problem in your area?	Yes	68	11	79	0.04	69.7–86.1
	No	15	6	21	0.04	14.0–30.2
What types of ticks are present in your area?	Hard tick	43	6	49	0.05	39.1–588
	Soft tick	20	2	22	0.04	14.8–31.3
	Both	20	9	29	0.50	20.8–38.7
Which type of ticks are most prevalent in cattle in your area?	Hard tick	70	11	81	0.40	71.9*–*87.6
	Soft tick	3	1	4	0. 02	1.4–10.3
	Both	10	5	15	03	9.2–23.5
Do you know the season when tick infestations occur in your area?	Yes	82	4	86	0.03	77.6–91.6
	No	1	13	14	0.03	8.4–22.4
In which season do tick infestations most commonly occur?	At the end of the rainy season	19	5	24	0.04	16.5–33.4
	At the beginning of the rainy season	8	1	9	0.02	4.6–16.6
	In mid of rainy season	5	4	9	0.02	4.6–16.6
	During the dry season	51	7	58	0.05	47.9–67.4
Are you aware of any tick-borne diseases?	Yes	80	9	89	0.03	81.1–93.8
	No	3	8	11	0.03	6.1–18.9
Which species of livestock are mostly infested by ticks?	Bovine	75	2	77	0.04	67.5–84.3
	Ovine	8	15	23	0.04	15.6–32.4
	Caprine	–	–	–	–	–
Which breed of cattle is more susceptible to tick infestations in your area?	Indigenous breed	5	2	7	0.02	3.3–14.1
	Crossbreed	13	5	18	0.03	11.5–26.9
	Exotic	65	10	75	0.04	65.4–82.6
Do you think the district veterinary clinic contributes to minimizing and controlling tick infestations?	Yes	59	2	61	0.05	50.9–70.1
	No	24	15	39	0.04	29.8–49.0
Have you ever used traditional medicine to control ticks?	Yes	83	17	100	0	–
	No	–	–	–	–	–
What types of traditional medicine do you use to control ticks?	Hot iron method	61	3	64	0.04	53.9–72.9
	Traditional plants	22	14	36	0.04	27.1–46.0

NB: % = Proportion; SE: Standard Error; CI = Confidence Interval.

## 4 Discussion

Ticks are well-known vectors of many animal diseases, making them an emerging economic and health burden in tropical and subtropical countries around the world (Taylor et al., 2015; Paucar et al., 2022). The overall tick prevalence in this study was 75.0 %. The high tick infestation level in this study means that ticks are an important external parasite of cattle in the study area. This could be attributed to livestock keepers' lack of awareness, poor cattle management, and environmental conditions, such as high temperatures, unpredictable precipitation, and humidity, that promote tick survival and reproduction (Kemal and Abera, 2017).

The current finding is consistent with the findings of Lemu et al. (2023) in Bedele district, Oromia Regional State, Tafesse and Amante (2019) in Guto Gida District, East Wollega Zone, Asefa et al. (2017) in Horo Guduru Wollega Zone, and Gedilu et al. (2014) in Bahir Dar, who reported the prevalences of 71.9 %, 71 %, 78.23 %, and 74 %, respectively.

Our findings were higher than the 25.6 % tick prevalence reported by Tiki and Addis (2011) in Holeta Town and the 34.3 % reported by Wondimu and Bayu (2021) in Haramaya. This variation could be attributable to changes in the research areas' agroclimatic conditions, sample collection season, sample size, and environment (Kemal et al., 2020). Tick activity can be affected by rainfall, height, and air relative humidity (Amante et al., 2014).

In this study, a total of 720 tick species were collected and identified. *R. evertsi evertsi* was the most abundant tick species, accounting for 38.7 % of all adult ticks collected. This is consistent with findings by Solomon et al. (2007), who described its widespread distribution over the Ethiopian faunal region. *A. variegatum* (25.5 %), *R. (Boophilus) decoloratus* (15.1 %), *A. lipidum* (12.5 %), and *H. rufipes* (8.1 %) ranked second through fifth in terms of abundance. The discrepancies in the collected ticks could be attributable to tick host preferences.

The variation in tick species prevalence in the study area can be attributed to several factors. The climatic conditions, characterized by high temperatures and erratic precipitation, create a conducive environment for different tick species. For instance, *R. evertsi evertsi* thrives in regions with warm temperatures and adequate humidity, which may explain its high prevalence (Tessema and Gashaw, 2010; Kemal and Abera, 2017). Seasonality also plays a crucial role, as certain tick species are more active during specific seasons (study period). For example, *A. variegatum* tends to peak in activity during the rainy season due to favorable moisture levels (Walker, 2003; Solomon et al., 2007; Kassa and Yalew, 2012). Understanding these dynamics allows for the development of targeted strategies to manage and reduce tick infestations effectively (Walker, 2003; Solomon et al., 2007).

Mamo Mezemir kebele had the highest tick burden, followed by Jitu Idris, Gadisa Godfo, and Abba Gada PAs. Furthermore, Mamo Mezemir and Jitu Idris *kebeles* were seventeen and two times more likely to be infested than Abba Gada kebele. This is most likely due to differences in animal owners' practices throughout the sampled *kebeles* to treat their animals. Strategic acaricide applications and rearing strategies may influence tick occurrence (Paucar et al., 2022).

The current study found that male animals were significantly more affected by ticks than females. These findings contrast those of Onu and Shiferaw (2013). This could be owing to differential exposure of males and females to tick infestations associated with their production and management conditions. Female animals, especially those used for milk production or breeding, often receive better nutrition and healthcare. They are typically monitored more closely and provided with superior shelter and hygiene practices to ensure their productivity and reproductive health (Mekonnen, 2001). This enhanced care can reduce their exposure to ticks and other parasites. In contrast, male animals, particularly those kept for purposes such as meat production, may not receive the same level of attention and care, increasing their vulnerability to tick infestations (Walker, 2003; Tashale and Merga, 2004). Additionally, sex hormones and genetic factors play a significant role in differential susceptibility to tick infestations. Research indicates that sex hormones such as testosterone in males can influence immune responses, potentially making males more susceptible to parasites. Testosterone has been shown to suppress certain immune functions, which could contribute to higher tick burdens in male animals. Conversely, females, with higher levels of estrogen, may have more strong immune responses that provide better defense against tick infestations (Choubdar et al., 2021; Fathi et al., 2024).

The current questionnaire survey revealed that 79 % of respondents recognized tick infestations in their area, with hard ticks notably affecting cattle production, including milk yield and growth, consistent with the findings of Kemal et al. (2016). Eighty-nine percent participants were aware of tick-borne diseases, and exotic and cross-bred cattle were found to be more susceptible to ticks than local breeds, aligning with the reports of Ayana et al. (2013) and Kemal et al. (2016). While 61 % of respondents noted the involvement of veterinary workers in tick control, no formal tick management plan exists in the study area, except for dairy farms (Mekonnen, 2001; Kemal et al., 2016). Respondents employed various control methods, with 64 % using hot iron and 36 % using traditional plants.

Livestock owners should adapt targeted acaricide applications and improve livestock management. There is also need to raise awareness about modern tick control methods, such as replacing ineffective traditional practices like hot iron branding (Paucar et al., 2022). Policy makers should strengthen veterinary services, implementing planned tick control strategies, and promoting educational programs tailored to community needs (Mekonnen, 2001; Kemal et al., 2016). Seasonal monitoring of tick activity should guide timely interventions during peak infestation periods, and encouraging the use of indigenous cattle breeds, which are more tick-resistant, can mitigate risks, especially in areas with limited veterinary support (Walker, 2003; Kemal et al., 2016). These findings emphasize the need for integrated approaches to tick control, combining environmental, management, and community-based interventions to reduce livestock losses and improve productivity, while ensuring sustainable, region-specific strategies (Solomon et al., 2007; Ayana et al., 2013).

The current study did not report tick distributions over the seasons. Furthermore, due to the short study period, the study only included cattle.

## 5 Conclusion

This study found that ticks are a prevalent and significant ectoparasite of cattle in the Toke Kutaye district, contributing to reduced productivity and health issues for livestock. The findings revealed that male cattle were more severely affected by ticks, with tick burdens varying across different areas. *R. eversti eversti* was found to be the most abundant tick species in the study area. The questionnaire survey indicated that all respondents were aware of tick infestations and reported using traditional control methods, such as hot irons and plants. Therefore, awareness creation for farmers and strategic and appropriate acaricide application are encouraged. In addition, further comprehensive studies on the overall season and the various tick species' roles in infesting cattle and other animals are recommended.

## CRediT authorship contribution statement

**Kirubel Abreham, Asemamaw Alemayew, Dimamnesh Worku**, and **Tefera Tarekagne** contributed to conceptualization, data collection, laboratory work, and manuscript draft; **Isayas Asefa Kebede** was involved in conceptualization, questionnaire development, resources, supervision, validation, write-up, re-editing, references searching, and reviewing; **Megersa Akasa Debele** contributed to questionnaire development and reviewing; **Segni Bedasa Gudina, Abrahim Dawed Ahmed**, and **Haben Fesseha Gebremeskel** were involved in the reviewing. All authors have approved the submission of the manuscript.

## Funding statement

No funding was received for this research.

## Declaration of competing interest

All authors declare no competing conflicts of interest.

## Data Availability

All the datasets generated or analyzed during this study are included in this manuscript.
